# Dr. Charles Parry

**Published:** 1861-09

**Authors:** 


### NECROLOGICAL NOTICES.

The medical profession of Indiana has just sustained a most serious loss in the death of DR. CHARLES PARRY, who expired a few days ago at his residence at Indianapolis. He was a graduate of the Jefferson Medical College of this city, and was widely known as a practitioner of medicine and surgery. He was a man of strong intellect and of excellent moral character. Dr. Parry contributed several valuable papers to the periodical press, distinguished by much vigor of thought, good taste, and admirable practical sense.

